# Increased MIB-1 Labeling Index Is Associated with Abducens Nerve Morbidity in Primary Sporadic Petroclival Meningioma Surgery: Beyond Location and Approach

**DOI:** 10.3390/curroncol29070398

**Published:** 2022-07-16

**Authors:** Johannes Wach, Tim Lampmann, Ági Güresir, Hartmut Vatter, Ulrich Herrlinger, Albert Becker, Marieta Toma, Michael Hölzel, Erdem Güresir

**Affiliations:** 1 Department of Neurosurgery, University Hospital Bonn, 53127 Bonn, Germany; tim.lampmann@ukbonn.de (T.L.); agi.gueresir@ukbonn.de (Á.G.); hartmut.vatter@ukbonn.de (H.V.); erdem.gueresir@ukbonn.de (E.G.); 2 Division of Clinical Neurooncology, Department of Neurology and Centre of Integrated Oncology, University Hospital Bonn, 53127 Bonn, Germany; ulrich.herrlinger@ukbonn.de; 3 Department of Neuropathology, University Hospital Bonn, 53127 Bonn, Germany; albert_becker@uni-bonn.de; 4 Institute of Pathology, University Hospital Bonn, 53127 Bonn, Germany; marieta.toma@ukbonn.de; 5 Institute of Experimental Oncology, University Hospital Bonn, 53127 Bonn, Germany; michael.hoelzel@ukbonn.de

**Keywords:** abducens nerve, MIB-1, petroclival meningioma, retrosigmoid approach

## Abstract

Abducens nerve palsy is a severe dysfunction after petroclival meningioma (PC MNG) surgery. The objective of this investigation was to analyze abducens nerve outcomes in patients who underwent the retrosigmoid approach in relation to the MIB-1 index. Thirty-two patients with primary sporadic PC MNG were retrospectively analyzed. Mean follow-up was 28.0 months. Analysis of the MIB-1 index was performed to evaluate the abducens nerve outcome. An optimal MIB-1 index cut-off value (<4/≥4) in the association with postoperative CN VI palsy was determined by ROC analysis (AUC: 0.74, 95% CI: 0.57–0.92). A new-onset CN VI palsy was present in 7 cases (21.88%) and was significantly associated with an increased MIB-1 index (≥4%, *p* = 0.025) and a peritumoral edema in the brachium pontis (*p* = 0.047) which might be caused by the increased growth rate. Tumor volume, cavernous sinus infiltration, auditory canal invasion, and Simpson grading were not associated with new CN VI deficits. Six (85.7%) of the 7 patients with both an increased MIB-1 index (≥4%) and new abducens nerve palsy still had a CN VI deficit at the 12-month follow-up. A peritumoral edema caused by a highly proliferative PC MNG with an elevated MIB-1 index (≥4%) is associated with postoperative abducens nerve deficits.

## 1. Introduction

Meningiomas are predominantly benign central nervous system (CNS) tumors, and they account for 36.4% of all CNS neoplasms [1,2]. Gross total resection with preservation of neurological functioning is suggested as the benchmark treatment for World Health Organization (WHO) grade 1 and 2 meningiomas [3,4]. Petroclival meningiomas (PC MNG) represent one of the most challenging meningiomas, and account for 5 to 11% of all posterior fossa meningiomas and only 0.15% of all intracranial tumors [5,6,7,8].

Despite the achievements of modern neurosurgery with the pursuit of a maximum of safety, surgical morbidities including cranial nerve deficits remain a tremendous complication resulting in further medical interventions and decreased quality of life [9,10]. Abducens nerve palsy is a frequently observed nerve dysfunction, and previous series reported incidences of new-onset palsies after surgery in up to 38.7% of patients [11,12,13,14]

To date, there are no molecular markers predicting patient-specific cranial nerve functional outcome. The Molecular Immunology Borstel-1 (MIB-1) is an immunohistochemical method to detect the Ki-67 antigen which is solely present in proliferating cells of the meningioma tissue [15,16]. Hence, the detection of this antigen enables the determination of the proliferative activity and provides a risk stratification regarding progression-free survival [17,18,19,20]. While tumor location and approach are established risk factors of postoperative cranial nerve dysfunctions, the impact of proliferative activity and inflammatory micromilieu in a cohort of patients who were homogeneously treated via the retrosigmoid approach only is unknown.

In a recent institutional series, we found that an increased MIB-1 index correlates with location-specific symptoms in meningioma (e.g., seizure burden in convexity meningiomas) [21]. However, the clinical influence of the MIB-1 index on abducens nerve outcome is unknown.

Therefore, we analyzed a homogeneous institutional population of 32 primary sporadic PC MNG patients who underwent surgical resection via a retrosigmoid approach in order to analyze potential factors influencing abducens nerve outcomes.

## 2. Materials and Methods

### 2.1. Study Design and Patient Characteristics

**Figure 1:**
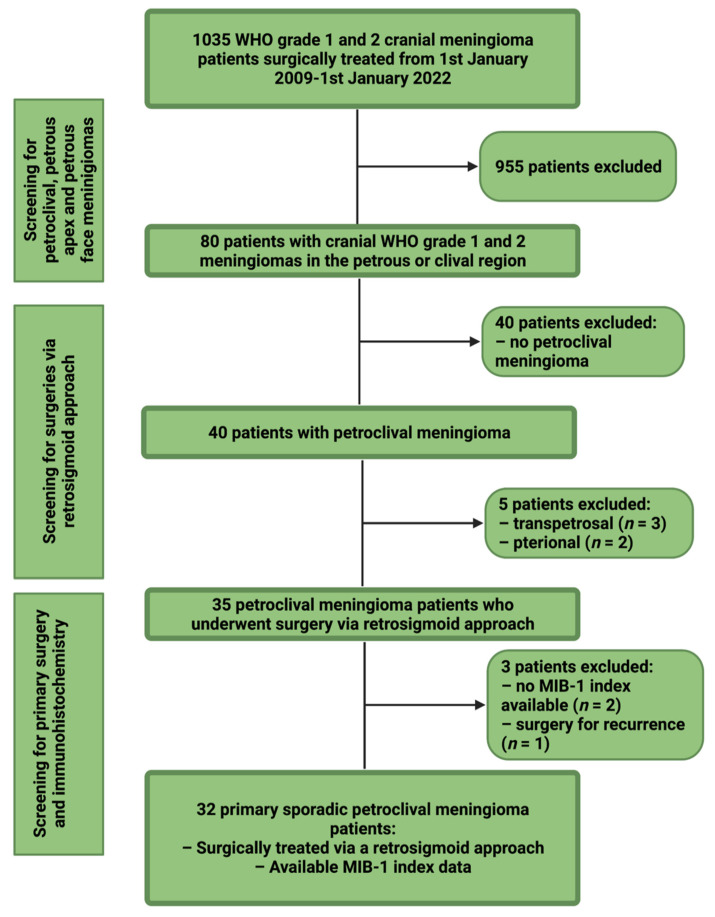
Flow chart illustrating the selection process of consecutive meningioma patients between 1 January 2009 and 1 January 2022.

Between January 2009 and January 2022, 1035 patients underwent surgical treatment for cranial WHO grade 1 and 2 meningioma at the institutional neurosurgical clinic. Retrospective evaluation of patient data was performed after approval by the institutional review board. Inclusion criteria of this investigation were neuropathologically confirmed meningiomas, petroclival localization, an age greater than 18 years, treatment via a retrosigmoid approach, surgery for primary sporadic meningioma, and the availability of the MIB-1 index. Patients with a neurofibromatosis type 2 associated meningioma or patients who underwent a prior radiotherapy were excluded because of their different histopathology and proliferative activity [22]. Thirty-two patients were included for the final study cohort (see Figure 1).

### 2.2. Data Recording

**Figure 2:**
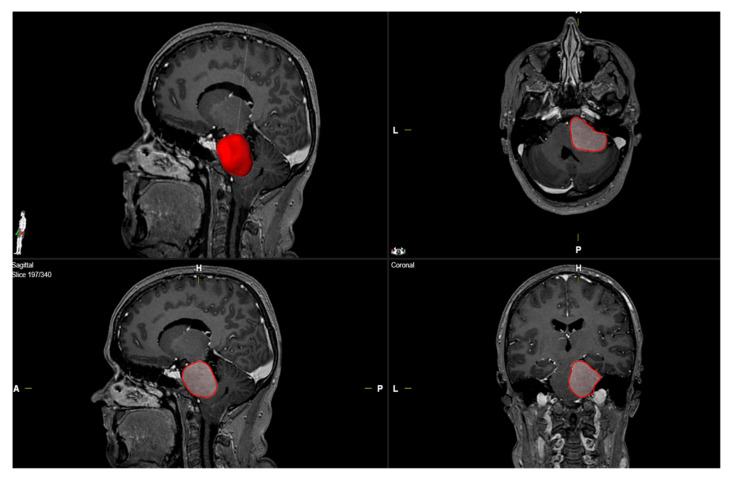
Illustrative determination of the tumor volume (red) in a female patient with a right-sided petroclival meningioma using SmartBrush (Brainlab Elements, Brainlab AG, Feldkirchen, Bavaria, Germany).

Clinical characteristics including age, sex, cranial nerve deficits, neurological functioning, body mass index (BMI), Karnofsky Performance Status (KPS), WHO grading based on neuropathological examination, extent of tumor resection based on the Simpson grading system in line with European Association of Neuro-Oncology (EANO) (Simpson grade 1–3 constitutes gross total resection, Simpson grade 4 constitutes subtotal resection, and Simpson grade 5 constitutes biopsy), and postoperative follow-up data were collected and entered into a computerized data sheet (SPSS, version 27 for Mac, IBM Corp., Armonk, NY, USA) [23]. Abducens nerve functioning was classified using the Scott and Kraft Score [24] (complete paralysis constituting a score of 1–2, incomplete paralysis constituting a score of 3–5, normal functioning constituting a score of 6). Laboratory markers were recorded using the laboratory information system Lauris (version 17.06.21, Swisslab GmbH, Berlin, Germany). Venous blood samples were routinely examined within 24 h prior to surgical treatment of PC MNG. These laboratory examinations were performed at constant time points, which enable a reliable analysis of the clinical endpoints. The routine blood examination protocol before surgical therapy included complete blood count, kidney test, liver tests, and the coagulation profile (INR, aPTT). The serum C-reactive protein values were determined by turbidimetric immunoassays with a CRPL3 reagent (Roche, Basel, Switzerland) [25]. Magnetic resonance (MR) imaging was performed in all cases within 48 h before surgical treatment. Peritumoral edema was defined as a hyperintense signal intensity adjacent to the tumor on T2-weighted MR-images [26]. MR-images were further investigated regarding the infiltration of the cavernous sinus, internal auditory canal invasion, and brainstem compression. PC MNG volume was calculated using 3D semi-automatic volumetry by the selecting the region of interest (Smartbrush^®^ software by Brainlab AG, Feldkirchen, Bavaria, Germany). Meningioma volumes were calculated by selecting the enhancing lesion in the T1-weighted MR-images after gadolinium injection (see Figure 2).

### 2.3. Institutional Treatment Strategy for Petroclival Meningiomas

PC MNGs were mainly evaluated and classified by their anatomy regarding origin of dural attachment, growth pattern, involvement of the Meckel’s cave, cavernous sinus and the internal auditory meatus. Institutional selection process of the approach of choice considers mainly the four recently described anatomical types of PC MNG [27]: Clivus type, petroclival type, petroclivosphenoidal type, and sphenopetroclival type. We prefer the retrosigmoid approach in different variations such as the traditional retrosigmoid approach, retrosigmoid transtentorial or retrosigmoid intradural suprametal approach for the following types of PC MNG: Clivus type, petroclival type, and petroclivosphenoidal type, which are predominantly in the posterior cranial fossa. Petroclivosphenoidal types of PC MNG, which invade into the middle cranial fossa were treated by a subtemporal transtentorial transpetrosal approach. Sphenopetroclival meningiomas which originate within the cavernous sinus or from the lateral wall of the cavernous sinus expanding into the parasellar region compromising the optic nerve or the cranial nerves III, IV, and VI were treated by an expanded pterional approach which predominantly results in a partial resection with the main goal of cranial nerve decompression. Institutional individual treatment scheduling regarding adjuvant radiotherapy is based on the extent of resection, WHO grade, and the patient’s performance status. Treatment algorithm is in line with the current recommendations of the EANO: Gross totally resected WHO grade 1 or 2 PC MNGs underwent a further observational regimen. Subtotally resected WHO grade 1 PC MNGs routinely undergo an observational management. Partially or biopsied WHO grade 1 PC MNGs with a substantial residual tumor mass undergo a fractionated stereotactic radiotherapy. All patients who do not undergo a gross total resection of a WHO grade 2 PC MNG are strongly recommended to perform a postoperative fractionated stereotactic radiotherapy if the postoperative performance status of the individual patient allows an adjuvant treatment [28]. New local tumor lesions or growing/progressive residual PC MNGs on a follow-up MRI examination were defined as meningioma progression [29].

### 2.4. Surgical Workflow

All patients in the present investigation underwent tumor resection using the retrosigmoid approach. A curved skin incision was made behind the ear. A retrosigmoid craniotomy was performed for the exposure of the connection between the sigmoid and transverse sinus. Afterwards, a curved incision along the inferior border of the transverse sinus and just posterior to the sigmoid sinus was made in the dura mater. Intraoperative electromyogram (EMG) monitoring of the facial and trigeminal nerve was performed in all cases. Electromyographic responses of the orbicularis oculi and oris muscles were used to monitor the facial nerve. Bipolar stimuli with an intensity of 0.05 to 1 mA and a duration of 0.1 ms were used. Furthermore, motor and somatosensory evoked potentials were routinely recorded. Monitoring of the lower cranial nerve groups were decided for each individual case and performed if deemed necessary. After the dural opening, cerebrospinal fluid (CSF) was slowly aspirated from the cerebellomedullary cistern to facilitate maximum safe microscopic surgery using the PENTERO 800 microscope (Zeiss, Oberkochen, Germany). Further workflow was as previously described [30].

### 2.5. Neuropathology

Neuropathological classification was performed based on the 2016 WHO criteria [3]. All histopathological reports underwent repeated examination to confirm that diagnosis was in line with these requirements. Immunohistochemistry was performed in the same workflow as described before for paraffin-embedded biopsy tissue specimen [31,32]. The MIB-1 labeling index was investigated using the following antibody: Anti-Ki67 (Clone 2B11 + PD7/26). Moreover, semiquantitative analysis and scoring of the CD68^+^ stainings using anti-CD68 antibodies to detect macrophages were performed (Clone KP1, dilution 1:1000, DAKO, Glostrup, Denmark). As previously described, meningioma specimens were investigated for the absence, focal or diffuse staining of CD68^+^ macrophages [33]. Visualization was performed using diaminobenzidine and a neuropathological examination was performed by expert neuropathologists including A.J.B.

### 2.6. Statistical Analysis

Data were organized and analyzed using SPSS for Mac (version 27.0; IBM Corp., Armonk, NY, USA). Kolmogorov–Smirnov (KS) test were performed to compare distributions. Normally distributed data are reported as the mean with the standard deviation (SD). Receiver-operating characteristic (ROC) curves were created to investigate the diagnostic performance of the MIB-1 index in the association with new-onset of abducens nerve palsy following PC MNG surgery via retrosigmoid approach. Cut-off values for the MIB-1 index were determined based on the ROC analysis. Preoperative demographics, tumor volume, tumor growth pattern, histopathological features, and immunohistochemical characteristics were compared between the patients with or without a new postoperative abducens nerve palsy using Fisher’s exact test (two-sided) for categorical data and independent *t*-test for continuous data. Receiver-operating characteristic curves were also created to display the correlation between MIB-1 index and perilesional edema as well as MIB-1 index and tumor progression in PC MNG. Kaplan–Meier charts were created to investigate the role of MIB-1 labeling index regarding local petroclival meningioma progression. Statistical results of the log-rank test were determined. A *p*-value of <0.05 was defined as statistically significant.

## 3. Results

### 3.1. Baseline Patient Characteristics

Thirty-two patients with a median age of 56 years (IQR 46–70) were enrolled in the present investigation. This analysis included 25 females (78.1%) and 7 males (21.9%; female/male ratio 3.6:1). Median (IQR) KPS was 90% (80-90). Median primary tumor volume (25th–75th percentile) was 13.0 cm^3^ (8.0–30.1). The most common preoperative symptom was compression of the trigeminal nerve (12/32; 37.5%) causing facial pain, numbness or paresthesia, followed by abducens nerve palsy (8/32; 25.0%), and symptoms of vestibulocochlear nerve disturbance (8/32; 25.0%). Patient and tumor specific characteristics are detailed in Table 1. Patients with a preoperative abducens nerve palsy had a mean (±SD) MIB-1 labeling index of 5.1 ± 4.1, whereas those without a preoperative abducens nerve dysfunction had a mean MIB-1 labeling index of 3.8 ± 1.9 (independent *t*-test: *p* = 0.23). Peritumoral edema in those with a baseline abducens nerve palsy was present in one patient (1/8; 12.5%), and those without a baseline CN VI palsy had a peritumoral edema in 7 cases (7/24; 29.2%), respectively (Fisher’s exact test (two-sided): *p* = 0.64). Mean tumor volume (± SD) in those patients with a preoperative CN VI palsy was 21.8 ± 13.3 cm^3^, and 17.3 ± 12.4 cm^3^ in those without a preoperative CN VI palsy, respectively (independent *t*-test: *p* = 0.41). Further baseline patient characteristics were also not associated with an already preoperatively existing sixth CN palsy.

### 3.2. Neuropathological Grading, Extent of Resection, and Postoperative Functioning

Tumor classification according to the WHO classification criteria included 28 patients with grade 1 (87.5%), and 4 patients with grade 2 (12.5%). Median (IQR) MIB-1 labeling indices in primary sporadic petroclival meningioma was 4.0 (3.0–5.0). Mean (± SD) MIB-1 labeling index was 4.2 ± 2.6. Mean (± SD) MIB-1 labeling index in WHO grade 1 PC MNGs was 3.6 ± 1.3, and mean (± SD) MIB-1 in WHO grade 2 meningiomas was 8.0 ± 5.6, respectively (independent *t*-test: *p* = 0.22). Diffuse infiltration of CD68^+^ macrophages was observed in 16 (50.0%) cases. With regard to the extent of meningioma resection, Simpson grade I&II resections were performed in 12 patients (37.5%), whereas 18 (56.25%) patients underwent Simpson grade III resection, and Simpson grade IV resection was performed in two cases (6.25%). The most common new-onset cranial nerve deficits after PC MNG surgery were a dysfunction of the abducens nerve (7/32; 21.9%). Six of the 7 patients with a new-onset abducens palsy had a complete paralysis (Scott and Kraft score of 1 & 2). Those six patients still had an abducens palsy at the 3-month follow-up, and three of those six patients recovered to an incomplete paralysis (Scott and Kraft score of 3–5) at 12-month, 24-month, and 36-month follow-up visit. One of the 7 patients with a new-onset abducens palsy after PC MNG surgery had an incomplete paralysis (Scott and Kraft Score of 5) at discharge, and at 9-month follow-up examination the patient had a normal abducens nerve functioning (Scott and Kraft score of 6). Further characteristics are displayed in Table 1.

### 3.3. Diagnostic Performance of the MIB-1 Index in the Association with Postoperative Abducens Nerve Palsy

**Figure 3:**
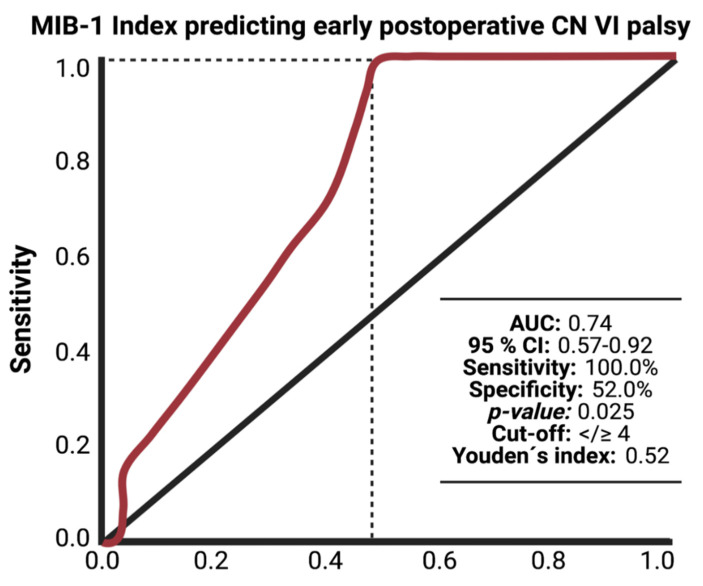
Receiver-operating characteristic curve (ROC) illustrating the ability of the MIB-1 index in the association with new postoperative abducens nerve deficits after surgery for primary sporadic petroclival meningioma via the retrosigmoid approach.

The MIB-1 index was available in all patients of the study cohort. The Median (IQR) MIB-1 labeling indices in primary sporadic petroclival meningioma was 4.0 (3.0–5.0). A ROC curve was constructed, and the AUC of the MIB-1 index in the association with new postoperative abducens nerve palsy after PC MNG surgery was calculated. The AUC of the MIB-1 index ROC curve for new postoperative abducens nerve palsies was 0.74 (95% CI: 0.57–0.92, *p* = 0.025). Sensitivity and specificity of the MIB-1 index in the association with new abducens nerve palsy were 100.0 and 52.0%, respectively (Youden’s index: 0.52), with a threshold set at ≥4%. Figure 3 shows the ROC curve and the results of the analysis. In total, 19 (19/32, 59.4%) had a MIB-1 index of ≥4%, and 13 (13/32; 40.6%) PC MNG patients had a MIB-1 index of <4%.

Patients with an increased MIB-1 index (≥4%) had significantly more often a peritumoral edema. Eight (8/19; 42.1%) of the patients with an increased MIB-1 labeling index had a peritumoral edema, whereas no peritumoral edema was observed in those cases (0/13; 0.0%) with a MIB-1 index <4% (Fisher’s exact test (two-sided): *p* = 0.01). Further clinical characteristics (BMI, KPS), inflammatory laboratory features (serum c-reactive protein, white blood cell count), tumor morphology (tumor volume, cavernous sinus infiltration, auditory canal invasion, brainstem compression), neuropathological characteristics (WHO grading, CD68 staining), and extent of resection (Simpson grading) were homogeneously distributed among the MIB-1 indices groups. Table 2 summarizes those results.

### 3.4. Association between New-Onset Abducens Nerve Palsy and Clinical, Tumor Morphology, and Neuropathological Characteristics

In total, 7 (21.88%) patients had a new-onset abducens nerve palsy, and 25 patients (78.12%) had a normal postoperative functioning of the abducens nerve after PC MNG surgery. Patients with a new-onset abducens nerve palsy had both significantly more often an increased MIB-1 index, and a peritumoral edema surrounding the PC MNG. All patients (7/7; 100%) with a new-onset abducens nerve palsy had an increased MIB-1 index (≥4%), and 48% (12/25) of those without an abducens nerve palsy had an MIB-1 index (≥4%), respectively (*p* = 0.025). Four (4/7; 57.1%) patients with a postoperative new-onset abducens nerve palsy had a peritumoral edema, whereas only 16% (4/25) of those without an abducens nerve palsy had peritumoral edema (Fisher’s exact test (two-sided): *p* = 0.047).

Tumor volume, growth pattern characteristics (cavernous sinus infiltration, internal auditory invasion, brainstem compression), clinical characteristics, and neuropathological features were homogeneously distributed among those patients with or without new postoperative abducens nerve deficits. Table 3 summarizes the results of the analysis.

**Figure 4:**
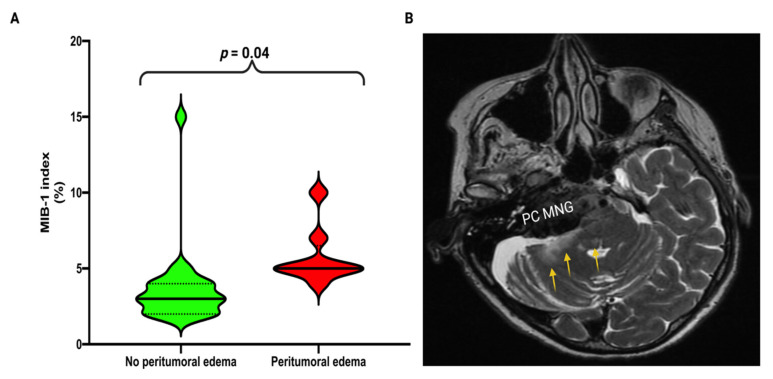
(**A**) Violin-plots displaying MIB-1 labeling index in patients without peritumoral edema (green) and with peritumoral edema (red) Violin plots show mean and distribution of MIB-1 index. Median values are presented by the thick black lines. (*p*-values of the Student’s *t*-test) (**B**) Illustrative image shows a T2-weighted MR-image of a 45-year old woman with a right-sided petroclival meningioma (PC MNG) and a new abducens nerve palsy after PC MNG surgery. The yellow arrows mark the peritumoral edema which is located in the right brachium pontis as well as in the posterior part of the pons which includes the abducens nucleus. Histopathology revealed a MIB-1 index of 7%.

Mean (± SD) MIB-1 index in those patients with a preoperative peritumoral edema in the brachium pontis or brainstem was 5.75 ± 1.9, whereas the patients without a baseline peritumoral edema had a mean MIB-1 index of 3.6 ± 2.6 (Independent *t*-test: *p* = 0.04). Figure 4 illustrate the significant association between increased MIB-1 index and peritumoral edema which results in an increased risk of a new postoperative abducens nerve palsy.

### 3.5. Perioperative Development of Preoperatively Existing Abducens Nerve Palsy after PC MNG Surgery

Two patients (2/8; 25.0%) of those with an abducens nerve palsy as presenting symptom had a regression of the abducens nerve dysfunction immediately after surgery (period until discharge) for sporadic PC MNG. Stable dysfunction was observed in 5 (5/8; 62.5%), and a progressive palsy was observed in only one case (1/8; 12.5%) after surgery. Mean (± SD) MIB-1 labeling index in those patients with a regression of the abducens nerve dysfunction after surgery was 3.0 ± 1.4, and in those with a stable or progressive already preoperatively existing dysfunction was 5.8 ± 4.5, respectively (*p* = 0.44). Baseline peritumoral edema was not present in those two cases with a regression of the preoperatively existing abducens nerve palsy, and the single case with a further progression of the preoperatively present CN VI palsy had a peritumoral edema. Mean tumor volume (± SD) in those patients with a regression of the preoperatively present CN VI palsy was 7.95 ± 7.1 cm^3^, and 26.35 ± 11.6 cm^3^ in those with a stable or progressive already preoperatively existing CN VI palsy, respectively (independent *t*-test: *p* = 0.09).

### 3.6. MIB-1 Index and Other New-Onset Cranial Nerve Palsies

The most common new-onset cranial nerve palsy after surgery was observed regarding the sixth cranial nerve. Further event rates of other postoperative new-onset cranial nerve palsies and their associations with MIB-1 labeling index as well as the presence of peritumoral edema were investigated. A statistical analysis using independent *t*-test comparing the mean (± SD) values of MIB-1 labeling indices in patients with or without other new-onset cranial nerve palsies was performed and identified no significant association. Furthermore, Fisher’s exact test (two-sided) was used to evaluate the association between peritumoral edema and new-onset palsies of other cranial nerves. Peritumoral edema was observed in all patients (2/2; 100%) of each group with either new-onset CN V palsy or CN VIII palsy, whereas peritumoral edema was present in only 22.2% of those patients without a new-onset palsy of the CNs V or VIII (Fisher’s exact test (two-sided): *p* = 0.06). Table 4 summarizes the results of those analyses.

### 3.7. MIB-1 Index and Probability of Progression-Free Survival after Petroclival Meningioma Surgery via Retrosigmoid Approach

**Figure 5:**
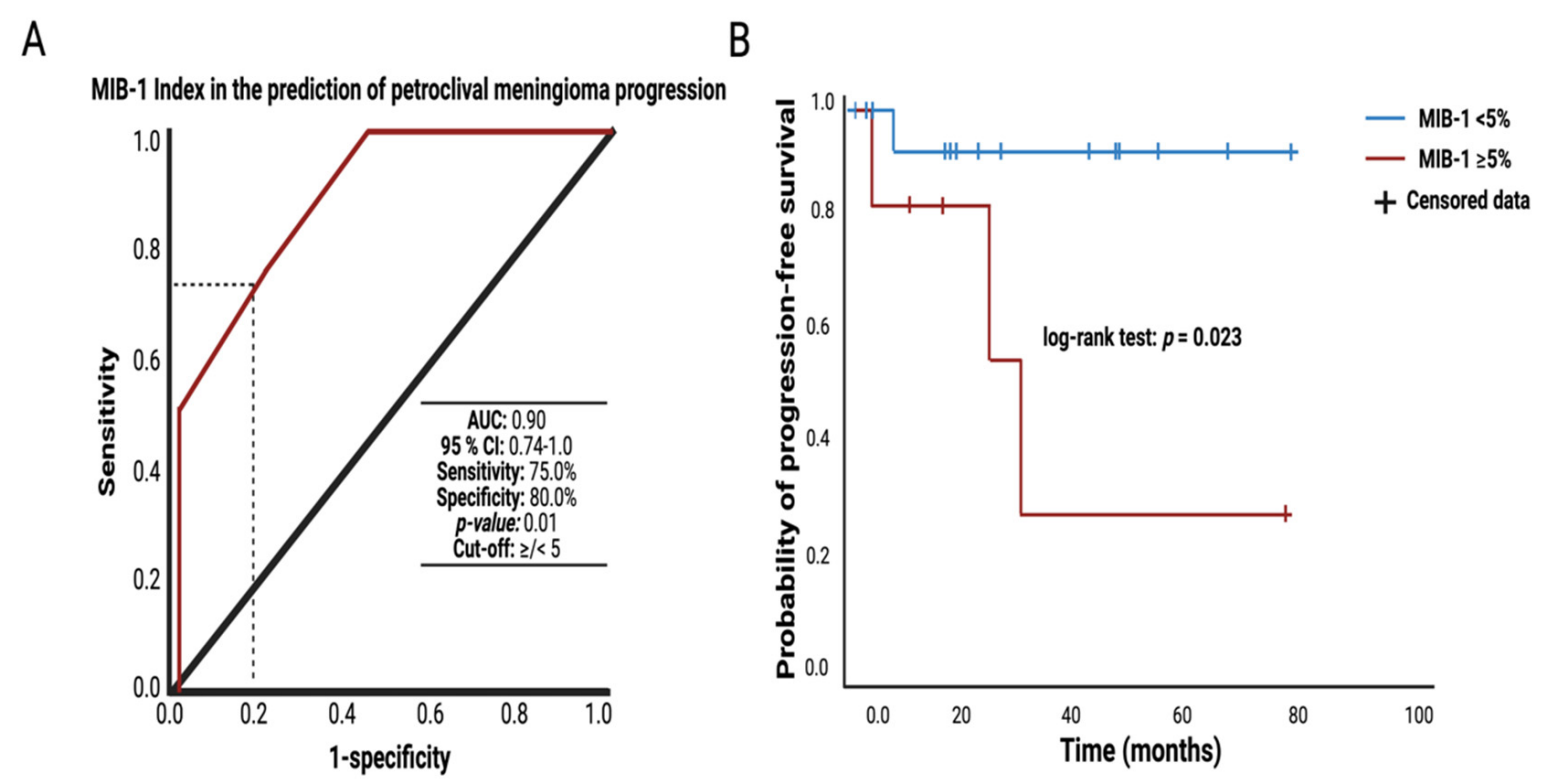
(**A**) Receiver-operating characteristic curve (ROC) illustrating the diagnostic power and the optimum cut-off value of the MIB-1 index in the association with petroclival meningioma progression after surgery for primary sporadic petroclival meningioma via the retrosigmoid approach. (**B**) Kaplan–Meier charts displaying the probability of progression-free-survival stratified by MIB-1 labeling index < 5% (blue line) and MIB-1 labeling index ≥ 5% (red line). Censored patients (stable disease at last follow-up) are indicated on the curves. The time axis right-censored at 100 months. *p* = 0.023 (Log-rank test).

Imaging follow-up was available in 26 (26/32; 81.3%) and the median (range) imaging follow-up was 21 (1–83) months. Four patients (4/26; 15.4%) were identified with a meningioma progression after surgical therapy. ROC curve analysis of the MIB-1 labeling index in the association with tumor progression was performed. The AUC was 0.90 (95% CI: 0.74–1.0). Optimum cut-off value of MIB-1 index in the association with tumor progression was identified at <5/≥5%. The sensitivity and specificity of the optimum threshold was 75.0% and 80.0%, respectively. The mean time to tumor progression in those cases with an increased MIB-1 labeling index (≥5%) was 39.6 (95% CI: 12.7–66.4) months, whereas those with an MIB-1 labeling index <5% had a mean time to tumor progression of 77.6 (95% CI: 67.5–87.8) months. The corresponding log-rank test (*p* = 0.023) revealed a significant association between MIB-1 labeling index and PC MNG progression. Figure 5 summarizes the results of the ROC curve analysis and the subsequent Kaplan–Meier analysis of MIB-1 index in the association with meningioma progression. Three progressive meningioma cases had an increased MIB-1 labeling index (≥5%) and underwent a Simpson grade III resection, whereas the fourth patient with a meningioma progression had no increased MIB-1 labeling index (<5%) and underwent a Simpson grade IV resection.

## 4. Discussion

The present study investigated potential variables being associated with new postoperative abducens nerve palsy after surgery via the retrosigmoid approach for primary sporadic WHO grade 1 or 2 petroclival meningioma. We found that an MIB-1 index of 5% or greater results in a perilesional edema of the brachium pontis as well as the pontine brainstem and is a risk factor for postoperative abducens nerve palsy.

Postoperative complications of the abducens nerve have been shown to be a frequent surgical morbidity of surgery for petroclival meningioma [34]. There is a high variety in the literature regarding the rate of new abducens nerve deficits following PC MNG surgery. The incidence in the literature ranges from relatively low rates at 5–8% to higher rates at 38.7% [11,12,13,14]. The incidence of new-onset abducens nerve palsy in the present investigation was 21.9% and ranks amongst the midfield regarding abducens nerve deficits compared to the reported rates. However, we could not observe the same recovery potential of the abducens nerve as described in a previous study Wang et al. [12]. In a prospective investigation of 31 petroclival meningioma patients they analyzed the outcome of the third and the sixth brain nerve after surgery using the anterior transpetrosal approach, orbitozygomatic approach or the presigmoid transpetrosal approach. They found that only two patients (2/12; 16.7%) had a residual abduction movement paralysis at the 6-month follow-up despite 12 patients suffering from new-onset abducens paralysis immediately after surgical resection of PC MNG. However, in our investigation all patients underwent the same surgical approach. Only one (1/7; 14.2%) of those patients with a postoperative new-onset abducens nerve palsy achieved a complete recovery of the abduction movement, whereas three (3/7; 42.9%) had a partial recovery within 36 months after surgery and the residual three patients (3/7; 42.9%) showed neither a recovery nor progression of the abducens nerve palsy. There are various theories debating the pathophysiology of abducens nerve dysfunction in PC MNG. For instance, an epidural invasion of the PC MNG around the Dorello’s canal in cases with infiltration of the cavernous sinus [34]. Furthermore, an encasement of the abducens nerve at the dural entrance as well as close adhesions between arachnoid membranes of the nerve’s cisternal portion and the meningioma are also discussed [35,36]. A recent retrospective study of 70 patients who underwent PC MNG surgery found that the invasion of the internal auditory canal is a risk factor for postoperative abducens nerve palsy [37]. Auditory canal invasion was present in 21.9% of all patients in our study cohort. However, we could not observe this PC MNG growth pattern as a risk factor for new postoperative abducens nerve dysfunction.

In our study, we have found a significant association between MIB-1 index values and the new-onset of a postoperative abducens nerve palsy. This association between the MIB-1 index and CN VI outcomes has not been described in previous studies. Furthermore, it was revealed that increased MIB-1 indices are correlated with a high density of macrophage infiltrates. In the present series we observed a trend that PC meningiomas exhibiting an increased MIB-1 index are overlapped by the simultaneous presence of dense CD68^+^ macrophage infiltrates. One explanation might be that local residual cells with increased proliferative potential driven by inflammation and tumor-associated macrophages disturb the local functioning of the abducens nerve by scar formation. This pathophysiological assumption might be the supported by the findings of Kato et al. [38]. They analyzed the correlation between cyclooxygenase-2 (COX-2) expression and the MIB-1 index in 76 meningioma cases. They found a strong correlation between COX-2 expression and MIB-1 index in all 76 cases. Furthermore, scar formation is a known part of prolonged inflammatory reactions, which is also induced by COX-2 expression [39]. Consequently, there might be a potential therapeutic target of non-steroidal anti-inflammatory drugs against the proliferative activity in meningioma to enhance progression-free survival as well as functional clinical endpoints such as cranial nerve functioning in skull base meningiomas. However, we also found that patients with a preoperative peritumoral edema in the brachium pontis and posterior part of the pontine brainstem had significantly more often a postoperative abducens nerve palsy. However, the development of this edema might be caused by the highly proliferative growth fractions of those meningiomas with an increased MIB-1 index. Several reports have shown a strong relationship between tumor aggressiveness and edema development in meningioma [17,40]. However, it has to be debated that the edema in the pontine brainstem in patients with an increased MIB-1 index and a new-onset postoperative abducens nerve palsy might also be caused by a disturbance of the abducens nucleus which is aggravated after the reveal of the compression by the tumor.

The present investigation has several limitations. The main limitation of this investigation is its retrospective nature, given the greater potential for missing variables regarding follow-up examinations. Moreover, there are some potential stumbling blocks as far as meningioma specimen sampling for MIB-1 index determination is concerned. Partially or subtotally resected meningiomas do not necessarily contain the “hotspot” area which represents the area of the tumor with the highest proliferative activity [41]. Furthermore, there is the potential of an interobserver variability regarding the investigation of the MIB-1 index in the present series due to the long time period which was analyzed. In addition, MIB-1 indices were not determined by a digital image software, which enables an analysis of a greater number of microscopic fields facilitating a more objective investigation [42]. Furthermore, the small sample size of the present study cohort did not allow a reliable multivariable analysis. However, we applied highly selective inclusion criteria and investigated only patients who underwent the retrosigmoid approach in order to reduce the potential confounding effect by including heterogenous surgical approaches in the analysis of new-onset abducens nerve palsies. Increased MIB-1 labeling indices might also affect the postoperative onset of other cranial nerve deficits. With the exception of the facial nerve as well as the lower cranial nerve group, all patients with other new postoperative cranial nerve deficits (CN groups: III, IV, V, VIII) had increased MIB-1 labeling indices compared to those patients without a new-onset palsy of other cranial nerves. However, the event rates of other cranial nerve deficits compared to the postoperative new-onset palsy of the abducens nerve were lower. Hence, those lower event rates of other postoperative new-onset cranial nerve palsies might have resulted that the univariable analysis revealed no significant association. Future multicentric trials analyzing a larger study cohort have to investigate our findings and might have a special focus on the residual cranial nerve groups.

In summary, the literature regarding MIB-1 index in sporadic meningiomas and our data might contribute to personalized surgical strategy planning. Future trials are needed to analyze the correlation between proliferative activity reflected by the MIB-1 index and COX2 expression in meningiomas. Furthermore, those trials might have a special focus on anti-inflammatory treatments improving postoperative outcomes. For instance, it was found that acetylsalicylic acid can facilitate the attenuation of demyelination and the thickening of myelin sheaths of axons during the regeneration process after a crush injury of the sciatic nerve [43]. Furthermore, there is emerging research on novel methods to intraoperatively determine the proliferative activity and inflammatory burden of neoplastic tissues. One method might be rapid immunohistochemistry using alternating current electric fields might be an option to enhance the interaction between antigens and antibodies. This novel approach enables a faster determination of the MIB-1 index and the proliferative potential intraoperatively [44,45]. Another interesting option to intraoperatively estimate the proliferative potential might be rapid flow cytometry which was found to be significantly correlated with the postoperative MIB-1 index in meningiomas [46]. Those methods might inform the surgical decision-making process regarding the weighting up the pros and cons of aggressive resection in terms functional preservation and provide a tailored therapy.

## 5. Conclusions

The present investigation is the first to demonstrate that high MIB-1 indices as a meningioma-intrinsic characteristic correlate with postoperative new abducens nerve deficits after surgery for petroclival meningioma. Furthermore, this finding might inform future trials modulating postoperative neuronal functioning in meningioma by anti-inflammatory treatment.

## Figures and Tables

**Table 1:** Patient characteristics (*n* = 32).

Median Age (IQR) (in Y)	56 (46–70)
Sex Female Male	25 (78.1%) 7 (21.9%)
BMI, Mean ± SD	26.5 ± 5.4
Median preoperative KPS (IQR)	90 (80–90)
Preoperative cranial nerve deficits CN II CN III CN IV CN V CN VI CN VII CN VIII Lower cranial nerve group	26 (81.3%) 5 (15.6%) 4 (12.5%) 1 (3.1%) 12 (37.5%) 8 (25.0%) 6 (18.8%) 8 (25.0%) 7 (21.9%)
Tumor volume, cm^3^, median (IQR)	$13.0\;{\mathrm{cm}}^{3}\;\left(8.0-30.1\right)$
Cavernous sinus infiltration	19 (59.4%)
Internal auditory canal invasion	12 (37.5%)
Peritumoral edema	8 (25.0%)
Brainstem compression	23 (71.9%)
Simpson grade Simpson grade I Simpson grade II Simpson grade III Simpson grade IV	3 (9.4%) 9 (28.1%) 18 (56.35) 2 (6.3%)
New-onset of cranial deficits CN II CN III CN IV CN V CN VI CN VII CN VIII Lower cranial nerve group	15 (46.9%) 0 (0%) 5 (15.6%) 2 (6.3%) 2 (6.3%) 7 (21.9%) 4 (12.5%) 2 (6.3%) 5 (15.6%)
WHO grade WHO grade 1 WHO grade 2	28 (87.5%) 4 (12.5%)
MIB-1 index, Median (IQR)	4.0 (3.0-5.0)
Brain invasion	0 (0%)
Mitotic figures	1.4 ± 1
CD68 staining Diffuse Focal none	16 (50.0%) 11 (34.4%) 5 (15.6%)

**Table 2:** Baseline clinical, laboratory, and imaging characteristics in petroclival meningioma patients with an increased and normal MIB-1 index (using Fisher’s exact test (two-sided) and independent *t*-test).

Characteristics	MIB-1 Index < 4% (13/32; 40.6%)	MIB-1 Index ≥ 4% (19/32; 59.4%)	*p*-Value
Age (years), mean ± SD	56.0 ± 14.2	58.6 ± 12.6	0.59
Sex Female Male	12 (92.3%) 1 (7.7%)	13 (68.4%) 6 (31.6%)	0.20
Body mass index, mean ± SD	26.2 ± 5.4	26.6 ± 5.6	0.84
Preoperative KPS, mean ± SD	86.2 ± 10.4	84.7 ± 9.6	0.70
Tumor volume, cm^3^, mean ± SD	14.2 cm^3^ ± 9.1	22.2 cm^3^ ± 14.1	0.09
Serum c-reactive protein, mean ± SD	5.3 ± 6.3	3.2 ± 4.3	0.22
White blood cell count, mean ± SD	6.5 ± 1.9	8.1 ± 2.7	0.07
Cavernous sinus infiltration Present Absent	6 (53.8%) 7 (46.2%)	13 (68.4%) 6 (31.6%)	0.28
Internal auditory canal invasion Present Absent	4 (30.8%) 9 (69.2%)	8 (42.1%) 11 (57.9%)	0.71
Peritumoral edema Present Absent	0 (0.0%) 13 (100.0%)	8 (42.1%) 11 (57.9%)	*0.01*
Brainstem compression Present Absent	8 (61.5%) 5 (38.5%)	15 (78.9%) 4 (21.1%)	0.43
Simpson grade <III ≥III	7 (53.8%) 6 (46.2%)	5 (28.6%) 14 (71.4%)	0.15
WHO grade 1 2	12 (92.3%) 1 (7.7%)	16 (84.2%) 3 (15.8%)	0.63
CD68 staining Diffuse Focal none	4 (30.8%) 7 (53.8%) 2 (15.4%)	12 (63.2%) 4 (21.1%) 3 (15.8%)	0.14

KPS = Karnofsky Performance Status; MIB-1 = Molecular Immunology Borstel-1; SD = Standard deviation.

**Table 3:** Comparison of patient characteristics in groups with or without new-onset abducens nerve palsy after semi-sitting surgery via retrosigmoid approach for primary petroclival meningioma (using Fisher’s exact test (two-sided) and independent *t*-test). *p*-values in italic represent statistically significant results.

Characteristics	No CN VI Palsy (25/32; 78.12%)	New-Onset CN VI Palsy (7/32; 21.88%)	*p*-Value
Age (years), mean ± SD	57.9 ± 14.1	56.4 ± 9.7	0.80
Sex Female Male	20 (80.0%) 5 (20.0%)	5 (71.4%) 2 (28.6%)	0.63
Body mass index, mean ± SD	26.6 ± 5.9	25.9 ± 3.9	0.75
Preoperative KPS, mean ± SD	84.4 ± 10.0	88.6 ± 9.0	0.33
volume, cm^3^, mean ± SD	17.8 cm^3^ ± 12.5	22.3 cm^3^ ± 17.6	0.87
Cavernous sinus infiltration Present Absent	14 (56.0%) 11 (44.0%)	5 (71.4%) 2 (28.6%)	0.33
Internal auditory canal invasion Present Absent	9 (36.0%) 16 (64.0%)	3 (42.9%) 4 (57.1%)	0.99
Peritumoral edema Present Absent	4 (16.0%) 21 (84.0%)	4 (57.1%) 3 (42.9%)	*0.047*
Brainstem compression Present Absent	18 (72.0%) 7 (28.0%)	5 (71.4%) 2 (28.6%)	0.99
Simpson grade <III ≥III	10 (40.0%) 15 (60.0%)	2 (28.6%) 5 (71.4%)	0.68
WHO grade 1 2	22 (88.0%) 3 (12.0%)	6 (85.7%) 1 (14.3%)	0.99
MIB-1 Index <4% ≥4%	13 (52.0%) 12 (48.0%)	0 (0.0%) 7 (100.0%)	*0.025*
Mitotic figures, mean ± SD	1.5 ± 2.3	1.1 ± 0.9	0.71
CD68 staining Diffuse Focal/none	12 (48.0%) 13 (52.0%)	4 (57.1%) 3 (42.9%)	0.99

KPS = Karnofsky Performance Status; MIB-1 = Molecular Immunology Borstel-1; SD = Standard deviation.

**Table 4:** Association between MIB-1 labeling index, and peritumoral edema with other new-onset cranial-nerve palsies (using independent *t*-test and Fisher’s exact test (two-sided)).

Cranial Nerves	Mean MIB-1 Index ± SD	*p*-Value	Peritumoral Edema	*p*-Value
CN III New-onset palsy (*n* = 5) Normal (*n* = 27)	4.20 ± 1.1 4.15 ± 2.8	0.97	2 (40.0%) 6 (22.2%)	0.58
CN IV New-onset palsy (*n* = 2) Normal (*n* = 30)	4.50 ± 0.7 4.13 ± 2.7	0.85	1 (50.0%) 7 (23.3%)	0.44
CN V New-onset palsy (*n* = 2) Normal (*n* = 30)	5.00 ± 0.0 4.1 ± 2.7	0.64	2 (100.0%) 6 (20.0%)	0.06
CN VII New-onset palsy (*n* = 4) Normal (*n* = 28)	4.00 ± 2.5 4.18 ± 2.7	0.90	2 (50.0%) 6 (21.4%)	0.25
CN VIII New-onset palsy (*n* = 2) Normal (*n* = 30)	5.00 ± 0.00 4.10 ± 2.7	0.64	2 (100.0%) 6 (20.0%)	0.06
Lower cranial nerve group New-onset palsy (*n* = 5) Normal (*n* = 27)	3.80 ± 1.3 4.22 ± 2.8	0.74	2 (40.0%) 6 (22.2%)	0.58

## Data Availability

The data presented in this study are available on request from the corresponding author. The data are not publicly available due to privacy and ethical restrictions.
